# Comparison of the gut microbiota and metabolism in different regions of Red Swamp Crayfish (*Procambarus clarkii*)

**DOI:** 10.3389/fmicb.2023.1289634

**Published:** 2023-12-22

**Authors:** Songyi Liu, Ziyan Wang, Ze Wang, Qiaoli Wu, Jun Zhou, Rixin Wang, Jiaojiao Han, Xiurong Su

**Affiliations:** ^1^State Key Laboratory for Managing Biotic and Chemical Threats to the Quality and Safety of Agro-Products, Ningbo University, Ningbo, China; ^2^School of Marine Science, Ningbo University, Ningbo, China

**Keywords:** *Procambarus clarkii*, metagenome, metabolites, intestinal microbiota, dominant bacteria

## Abstract

**Background:**

The gut microbiota is very important for maintaining the homeostasis and health of crustaceans. Many factors affect the gut microbiota of crustaceans, one of which is temperature. However, it is currently unclear how temperature affects the gut microbiota and metabolites of *Procambarus clarkii*.

**Methods:**

Using metagenomic sequencing and gas chromatography–mass spectrometry (GC–MS) techniques, the gut microbiota and metabolites of *P. clarkii* from Hubei (HB), Jiangsu (JS), Shandong (SD), and Zhejiang (ZJ) in China were investigated.

**Results:**

Under the impact of temperature, the gut microbiota and metabolites of *P. clarkii* exhibit a specific trend of change. The primary pathogenic bacteria affecting *P. clarkii* are *Citrobacter, Enterobacterium*, and *Aeromonas*, which are affected by temperature. Two metabolites, namely, sugars and amino acids, are regulated by temperature.

**Implication:**

This study demonstrated that the gut microbiota and gut metabolites of *P. clarkii* were considerably affected by temperature. It provides a theoretical basis for the systematic study of *P. clarkii* and provides a basis for a healthy culture of *P. clarkii*.

## Graphical Abstract

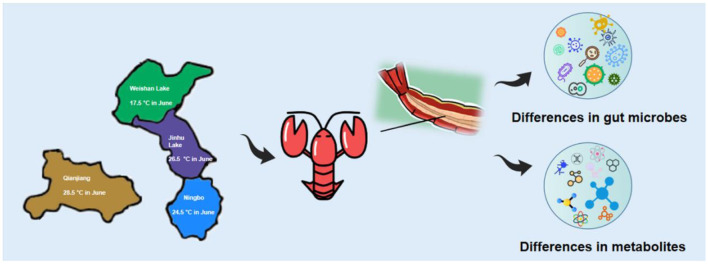

## Key points

- Gut microbiota and metabolites of *Procambarus clarkii* were compared for the first time from four representative habitats.
- There are effects of temperature on *Procambarus clarkii* metabolites and gut microbiota.
- *Procambarus clarkii* from various settings were compared using the combined study of their metabolites and gut microbiota.

## Introduction

Red swamp crayfish, *Procambarus clarkii*, belongs to an important group of crustaceans, characterized by its fast growth rate and strong adaptability (Zhong et al., 2021). *P. clarkii* is a popular freshwater economic species. It was enormously loved by consumers and used after it first came to China in the early 1980s (Yi et al., 2018; Yuan et al., 2021). In China, *P. clarkii* farming is primarily distributed in polders of plains, lakeshore areas, coastal areas, and the Pearl River Delta, including Jiangsu, Shandong, Zhejiang, and Hubei (Dong et al., 2022). Studies conducted on this species have encompassed heavy metal assessments, growth and reproduction, and water quality. However, limited knowledge is available regarding the regional influences on the metabolites and gut microbiota of *P. clarkii* (Kouba et al., 2021; Chen et al., 2022b; Mo et al., 2022). *P. clarkii* prefers hot and humid environments for growth, there are certain differences in the climate and other conditions of different production areas, and the microorganisms carried by *P. clarkii* after they come out of the pond are also different.

The peak season for *P. clarkii* starts in June, and the meat becomes very plump. June, July, and August are the months when *P. clarkii* has the strongest growth. *P. clarkii* is very susceptible to continuous high-temperature stress during summer breeding. For instance, in Hubei, the temperature in July presents an average of 32.94°C and instantaneous temperature of 36°C. Even higher temperatures are observed in August. *P. clarkii* thrive best in temperatures ranging from 21 to 28°C, and high-temperature stress can trigger a metabolite disturbance in *P. clarkii* (Guo et al., 2020). Therefore, this experiment was conducted at the beginning of the peak season of *P. clarkii*, when the temperature was moderate. *P. clarkii* inhabit an environment that varies considerably from area to region, and different climates have different temperatures. Jining, which is situated in the southwest of Shandong Province, is surrounded by the Huang-Huai-Hai and central mountains. The region is a typical monsoon one. In June, the average temperature in Jining was 17.5°C. Qianjiang is a unique city that may be found on the Jianghan Plain in the south-central region of Hubei Province, straddling the Yangtze and Han rivers. It is known as the “hometown of lobster” and “water garden.” It is a humid, subtropical northern monsoon region. June's average temperature was 28.5°C. The eastern portion of China's maritime area and the south wing of the Yangtze River Delta are where Ningbo City, Zhejiang Province, is situated. It is a typical Jiangnan water town and harbor city, and it has a subtropical monsoon climate. In June, the average temperature was 24.5°C. In the north-central region of Jiangsu Province, in the Yangtze River Delta, is where you can find Xuyi. Here, the fourth-largest freshwater lake in China, Hongze Lake, is connected to the Huaihe River's east and north. It has a humid monsoon climate, and the average temperature in June was 26.5°C. Temperature is a crucial component because it affects how animals function physiologically, especially in water, where their body temperature varies according to water temperature (Sepulveda and Moeller, 2020).

The gut microbiota is rich and contains bacteria, archaea, eukaryotes, and viruses (Li et al., 2023). In the lengthy evolutionary process, a stable state between the host and gut microbiota has developed (Bates et al., 2006). It has been reported that many factors affect the gut microbiota composition in the host, such as environmental factors, which are also key determinants of gut microbiota in many aquatic species (Pan et al., 2023). A study suggests that environmental temperature has a greater impact on the gut microbial diversity of *Cyprinus carpio* and *Micropterus salmoides* (Zhang et al., 2023). A recent study suggested that there were differences in the bacteria of two lizards (*Phrynocephalus erythrurus* and *Phrynocephalus przewalskii*) at different temperatures, which facilitated their environmental adaptation of the two species (Chen et al., 2022a). Similarly, large-scale sequencing of yak (*Bos grunniens*) feces from three different geographical regions demonstrated how the gut microbiota of yaks varied in both composition and variety (Liu et al., 2021). Most recent surveys have proved distinctions in the gut microbiota of zebrafish (*Danio rerio*) by analyzing feces (Wang et al., 2022a). This sequencing technique is a powerful instrument for studying gut microbiota (Reuter et al., 2015). Studies of gut microbiota patterns have mostly concentrated on microbial composition by high-throughput DNA sequencing, 16S rDNA, with relation to the ditchless and classic rice-crayfish (*Procambarus clarkii*) in the Sichuan basin (Huang et al., 2022). Compared with it, metagenomics can fully reveal the species of the microbiota of the farmed adult turbot (*Scophthalmus maximus*) and their functions and can greatly improve the speed and accuracy of sequence analysis (Xing et al., 2013). Studies on animal gut microbiota have drawn a lot of attention recently. Studying animal gut microbiota can not only analyze the function of its gut microbiota but also provide an understanding of the changes, signaling pathways, and response mechanisms of its gastrointestinal microbiota in both wild and artificially reared bar-headed goose (*Anser indicus*) (Wang et al., 2017). In addition, by tweaking the gut microbiota, some diseases can be effectively guarded against and treated, which is also beneficial to the health, sustainable reproduction, and survival of animals (*Apis mellifera*) (Masry et al., 2021). Furthermore, metabolomic research indicates that the gut microbiota of Beijing-You chicken and Cobb 500 broiler can promote the gut to produce amino acids, sugars, and other substances necessary for life processes (Chen et al., 2023). For now, metabolomics is widely applied in various studies, and it is widely used in research fields such as food and microbiota, such as targeted modulation of *Macrobrachium rosenbergii* gut microbes and metabolites, and improvement of intestinal function (Zheng et al., 2022). Researchers have discovered that *Litopenaeus vannamei*, which is raised in multiple aquaculture systems, exhibits different metabolite profiles via GC–MS technology (Suantika et al., 2020). GC–MS was employed to thoroughly examine the impact on the metabolism of male piglets with cold stress (Zhang et al., 2022). There are few reports on how temperature affects the structure and metabolites of gut microbial communities in *P. clarkii*. Therefore, it is necessary to further clarify the relationship between them.

A key factor shaping aquatic animal microbial communities is ambient temperature. Therefore, we hypothesized that altering temperatures would affect the organization of the gut microbiota of *P. clarkii*, thereby affecting metabolites or growth and physiology. The study's goal was to assess and evaluate abundance shifts, composition, and function to learn more about the basic structure and major microbial species found in *P. clarkii*'s gut microbiota at various ambient temperatures. In addition, intestinal metabolites of *P. clarkii* from different habitats were compared by GC–MS. These findings will aid in determining how the external environment affects the composition, functionality, and mechanism of the *P. clarkii* gut microbial population. The development of microbial control plans for aquaculture systems may benefit from these findings, serving as a guide for *P. clarkii* aquaculture that is healthy.

## Materials and methods

### Sample preparation

**Figure 1:**
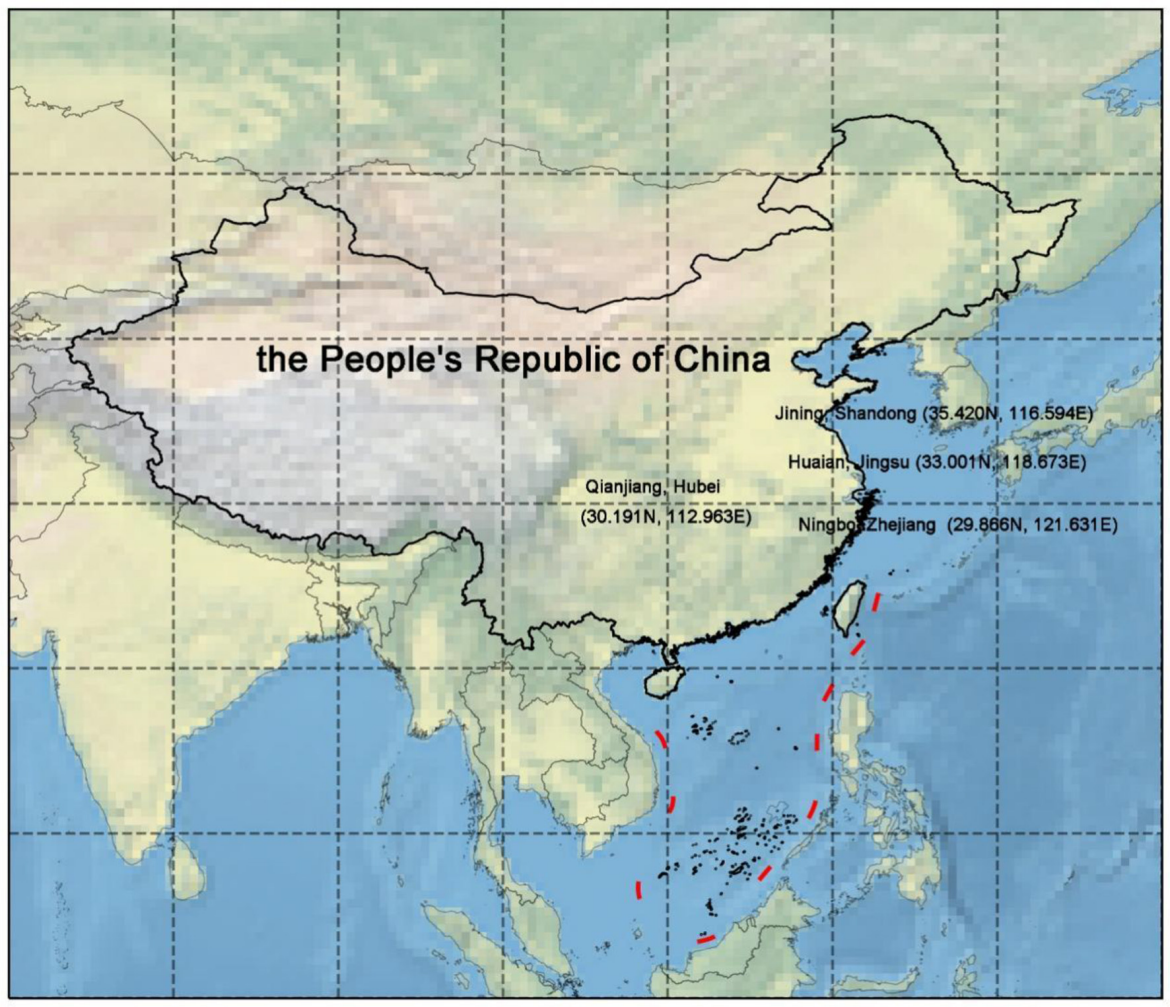
Location map. Quote the data of China's provincial administrative boundary in 2015 from the Resource and Environmental Science Data Center of the Chinese Academy of Sciences and MeteoAI (zhangqibot) provincial administrative boundary including nine segments.

In this study, four locations, namely, Weishan Lake in Jining (Shandong Province, China, SD), Qianjiang in Hubei Province (HB), Jinhu Lake in Xuyi (Jiangsu Province, China, JS), and Ningbo, Zhejiang Province (ZJ), were selected to serve as breeding grounds for *P. clarkii* from January to June. The water's parameters, such as pH 7.0–8.5 and dissolved oxygen > 5 mg/L, were all kept within a reasonable range. The optimal breeding density for *P. clarkii* was ~10–15/m^2^. *P. clarkii* was fed specific foods twice a day, adjusting the daily feeding amount according to the weight of *P. clarkii* and making sure the feed had a minimum of 30% crude protein content. By the end of June, *P. clarkii* was harvested, and samples of *P. clarkii* are currently fresh. The locations of these four places are shown in Figure 1. The transportation of the specimen was ensured at 4°C. For different indicator measurements, *P. clarkii* with unbroken shells, uniform sizes, and a mass of 40–50 g were chosen, excluding those that perished during shipment. In a germ-free environment, the intestinal contents of *P. clarkii* were collected. They were immediately placed in a sterile Eppendorf tube and immediately immersed in liquid nitrogen.

### DNA extraction and metagenomic sequencing

Microbial DNA has been obtained using the bead pounding and spinning screen technologies of the EZNA Soil DNA kit D5625-01 (Omega Bio-Tek, Inc., Norcross, GA, USA). Using the NanoDrop ND-1000 spectrometer (Thermo Fisher Scientific, Waltham, MA) and a gel of agarose electrophoresis (1%), the quantity and purity of DNA extraction were evaluated. The gathered DNA was processed using the Illumina TruSeq Nano DNA LT Library Preparation Kit to create payable-end 150 bp shotgun sequencing libraries with a put spanning 400 bp. Personal Biotechnology Co., Ltd. (Shanghai, China) sequenced each library via the Illumina HiSeq X-ten stage (Illumina, USA) and the PE150 process.

After the low-quality sequence and the sequencing adaptors were removed from the first data obtained by sequencing, species annotation was performed using Kraken2. Enabling high-precision and fast species annotation used precision short genomic substrings (k-mers) matching. MEGAHIT (v1.0.5) built *de novo* assembly using a concise de Bruijn graph (SdBG) technique. The default parameter was selected as meta-sensitive with MEGAHIT, used the default mode of single sample concatenation, combined unmapped reads, and concatenated them again. MetaGeneMark (http://exon.gatech.edu/GeneMark/) performed gene prediction and further clustered 95% protein similarities to obtain a non-redundant gene catalog. Contigs were obtained and synchronized with the NCBI-NT database (v2019.8.12, expectation value threshold: 0.00001). Each contig's taxonomical level was determined by applying Blast2lca's (https://github.com/emepyc/Blast2lca) minimal shared ancestor-based operational rule. The information on species classification at every level was collected. The α diversity and β diversity were calculated using QIIME2. To find relevant gene annotations, the contigs were compared to the KEGG database (http://www.genome.jp/kegg/) (Consultation, 2007; Sun et al., 2020).

### Metabolomic analysis by GC–MS

Each sample was precisely weighed to a 50 mg level and fitted in an Eppendorf tube measuring 1.5 mL. Following that, 400 μL PBS (pH = 7.4) was gained and oscillated for 2 min. The result solution was spun down under 13,000 rpm for 10 min after being oscillated for 10 min. The remaining samples were joined to 400 μL pre-cooled methanol and violently mixed. They were extracted for a while and centrifuged. The resulting two supernatants were oscillated and centrifuged again. After adding the appropriate amount of supernatant to a sample bottle and tetracosane standard solution, the liquid evaporated under a stream of nitrogen, take 50 μL of Meox, whose concentration is 20 mg/mL, oscillate, and metallic bath at 60°C with 1.5 h, and get 100 μL of MSTFA, oscillate, and metallic bath at 60°C for 1.5 h.

The GC–MS, 7890/M7-80EI (Agilent Technologies, Santa Clara, CA, USA; Beijing Persee General Instrument Co., Ltd., Beijing, China) was used for conduct analysis. Helium was carrier gas (purity not < 99.999%) at a flow of 1 mg/mL. The chromatographic column, Agilent DB-5MS (Agilent Technologies, Santa Clara, CA, USA), the specification of 30 m × 0.25 mm × 0.25 μm, was used for this experiment. The sample size was 1 μL, started at 60°C, and held for 3 min. The speed was 5°C/min up to 200°C, sustained for a moment. From 200°C to 300°C, it was held for 10 min. The input, transmission line, and ion source were all set at temperatures of 280°C, 250°C, and 280°C. With electron energy (70 eV) running in full scan mode (m/z 50-800), the separated proportion was 1:10 (Pechlivanis et al., 2014; Deda et al., 2018).

Mass spectrometry data processing procedures included baseline filtering, baseline calibration, peak comparison, deconvolution, peak recognition, and peak area integral. The metabolites were identified using the NIST database, and all accepted false-positive peaks were removed to provide a CSV file. The use of internal standard calibration helped to minimize variations between different samples. Multivariate statistical analysis helped to tentatively identify different metabolites.

### Statistical analyses

The mean and standard deviation are used to express every piece of data (m ± SD). The index of *p* < 0.05 is a statistical difference notably.

### Accession numbers

Sequences with accession numbers PRJNA952501 have been deposited in the NCBI Sequence Read Archive database.

## Results

### Diversity of the gut microbiota

**Figure 2:**
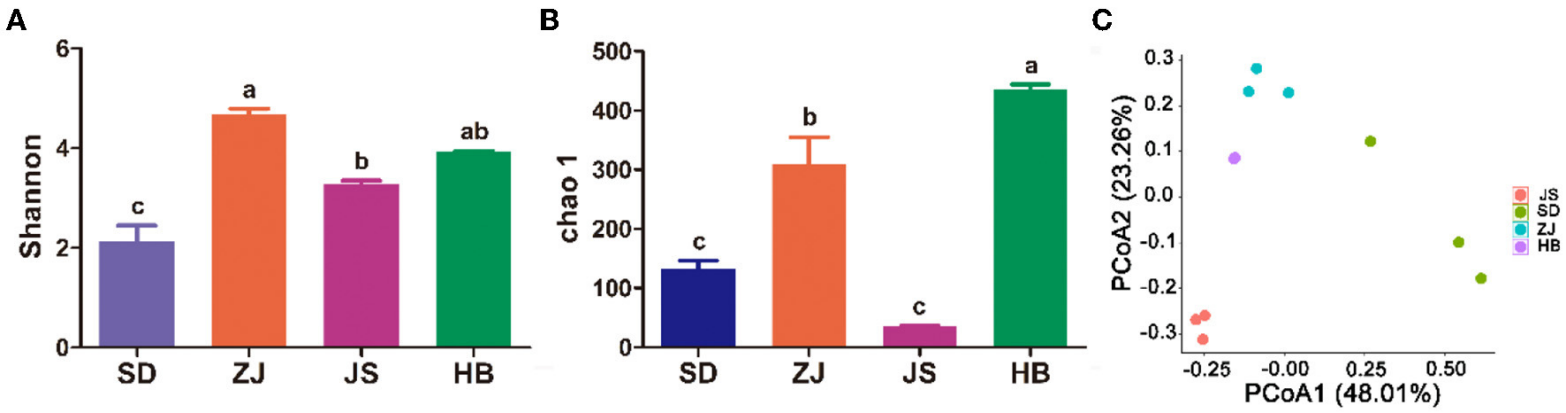
Diversity of the gut microbiota in *P. clarkii*. **(A)** Shannon index. **(B)** Chao1 index. **(C)** PCoA.

In this study, the Shannon and Chao1 indices were employed to calculate the gut microbiota's richness and diversity. In Figures 2A, B, among the other three groups, the ZJ group's Shannon index was in first place, indicating higher diversity (*p* < 0.05), but out of the four groups, the SD group had the least amount of diversity. The JS group was shown to be lower than the other groups since their Chao1 index was much lower than the other three groups (*p* < 0.05). The HB group exhibited taller richness, having a higher Chao1 index than other groups (*p* < 0.05), and the HB group had taller richness. Four groups were separated according to principal coordinate analysis (PCoA), showing that the microbial compositions varied. Multiple types of microbiota in the gut were found in each of the four groups, according to PCoA (Figure 2C).

### Diversity of the gut microbiota

**Figure 3:**
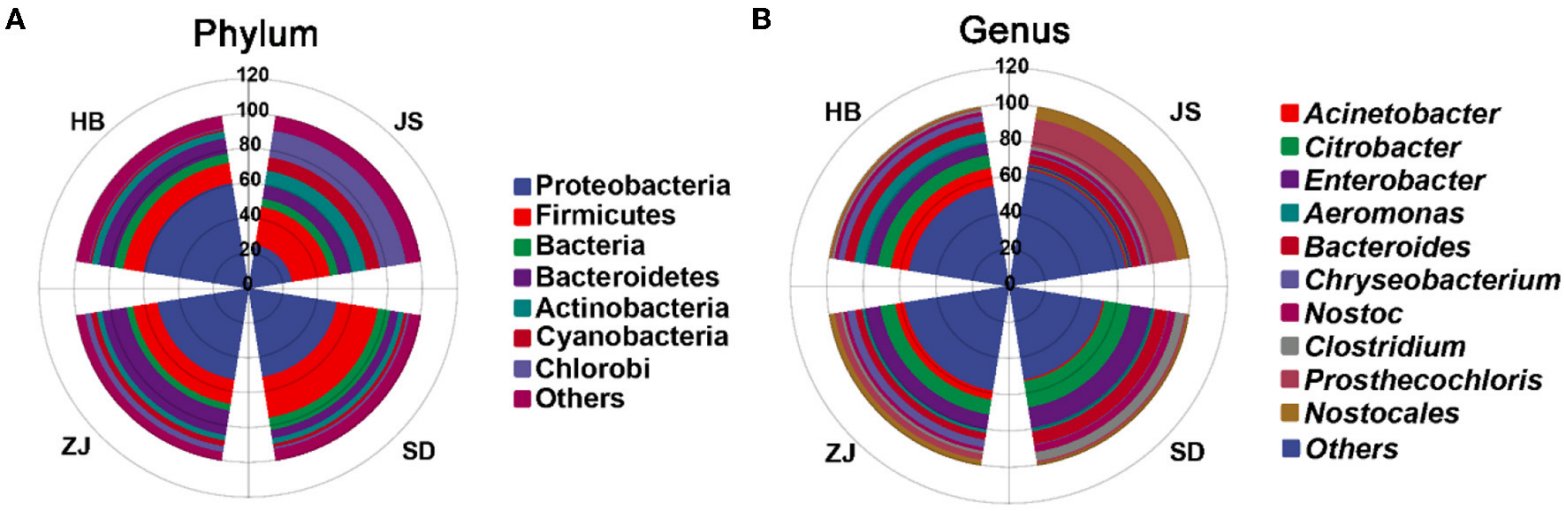
Distinct gut microbiota compositions in *P. clarkii*. **(A)** Phylum-level composition of the gut microbiota. **(B)** Genus-level composition of the gut microbiota.

In total, 50 bacterial phyla were explored in the JS, SD, ZJ, and HB groups, which mainly consisted of Proteobacteria, Firmicutes, Cyanobacteria, Bacteroidetes, Candidatus Saccharibacteria, and Chlorobi. Proteobacteria was most prevalent in the HB group (61.29 ± 0.11%), followed by the ZJ group (53.13 ± 9.40%), the SD group (51.55 ± 6.67%), and the JS group (25.36 ± 0.41%). In the HB group, the abundance of Chlorobi and Cyanobacteria was lower (0.59 ± 0.02% and 1.15 ± 0.09%, respectively). There were fewer Actinobacteria in the ZJ group (3.17 ± 0.66%). In the SD group, the abundance of Chlorobi was lower (1.21 ± 0.66%). In the JS group, there were fewer bacteria (4.74 ± 0.09%) (Figure 3A).

The microbiota in four groups, at the genus level, mainly comprised *Citrobacter, Enterobacter, Bacteroides, Acinetobacter, Aeromonas, Nostoc, Clostridium, Chryseobacterium*, and *Prosthecochloris*. *Acinetobacter* was more prevalent when compared to other categories (10.07 ± 0.03%), and the abundance of *Prosthecochloris* (0.59 ± 0.05%) was a little in HB. In ZJ, the magnitude of *Citrobacter* was more widespread in contrast to other categories (8.35 ± 3.43%), and the abundance of *Clostridium* (1.55 ± 0.15%) was lower. *Citrobacter* had the largest abundance in the SD group (14.71 ± 3.76%), while *Chryseobacterium* had a lower abundance (0.42 ± 0.11%). The abundance of *Prosthecochloris* was highest (15.38 ± 0.47%), and the abundance of *Aeromonas* (0.59 ± 0.03%) was lower in the JS group (Figure 3B).

### Functional analysis of gut microbiota

**Figure 4:**
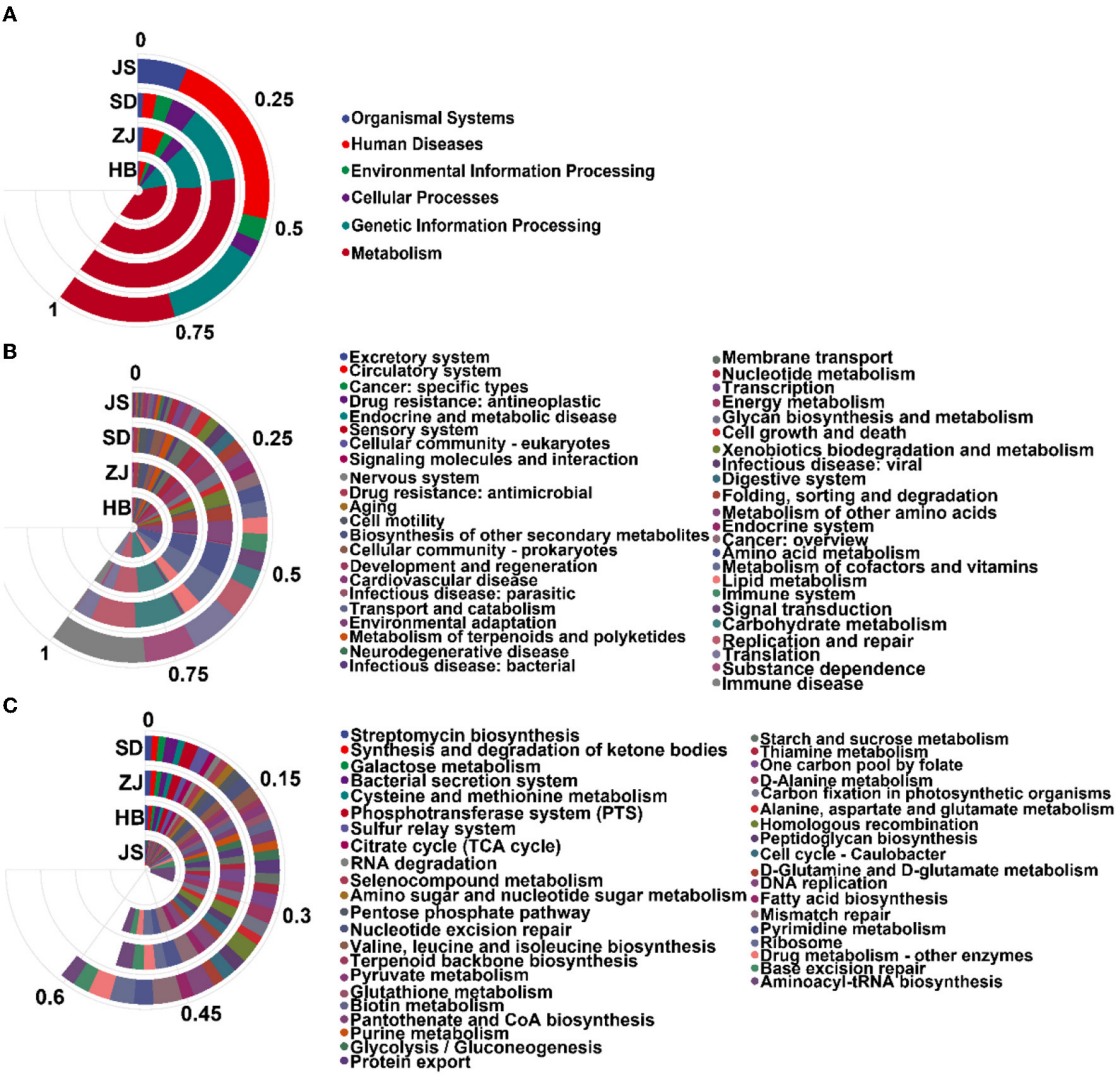
Analysis of the enrichment pathway of KEGG in *P. clarkii*. **(A)** The primary classification information of the KEGG pathway. **(B)** The secondary classification information of the KEGG pathway. **(C)** The third classification information of the KEGG pathway.

Relation to the microbiota, database, functional notes, and abundance information can be divided into six parts: metabolism, cellular processes, genetic information processing, environmental information processing, human diseases, and organismal systems. In HB, ZJ, SD, and JS, respectively, the pathway in metabolism accounted for 62.76 ± 0.22%, 59.51 ± 3.23%, 61.49 ± 0.90%, and 24.21 ± 0.45% of the identified pathways. In total, 11 paths of biochemistry were found. Compared to the other groups, the JS group performed dramatically worse in amino acid and carbohydrate metabolism. A majority of pathways involved in replication, repair, and translation were also seen in groups from each location (Figures 4A, B).

The KEGG database Pathway Entry was used to find the top 40 most prevalent pathways. In the JS group, aminoacyl-tRNA biosynthesis (8.11 ± 0.21%), base excision repair (3.41 ± 0.06%), and ribosome (3.6 ± 0.06%) were the most enriched pathways. In the SD group, the most enriched pathways were mismatch repair (3.64 ± 0.93%), ribosome (2.87 ± 0.15%), DNA replication (2.80 ± 0.67%), homologous recombination (2.54 ± 0.53%), and pyrimidine metabolism (2.49 ± 0.60%). The ZJ group had the highest enrichment of some pathways, including aminoacyl-tRNA biosynthesis (2.90 ± 0.90%), mismatch repair (2.79 ± 0.51%), pyrimidine metabolism (2.43 ± 0.52%), DNA replication (2.20 ± 0.48%), and homologous recombination (1.83 ± 0.29%). These pathways, including mismatch repair (2.778 ± 0.04%), ribosome (2.72 ± 0.03%), DNA replication (2.18 ± 0.03%), aminoacyl-tRNA biosynthesis (2.01 ± 0.03%), and Homologous recombination (2.01 ± 0.02%), were most enriched in the HB group (Figure 4C).

### Metabolite analysis

**Figure 5:**
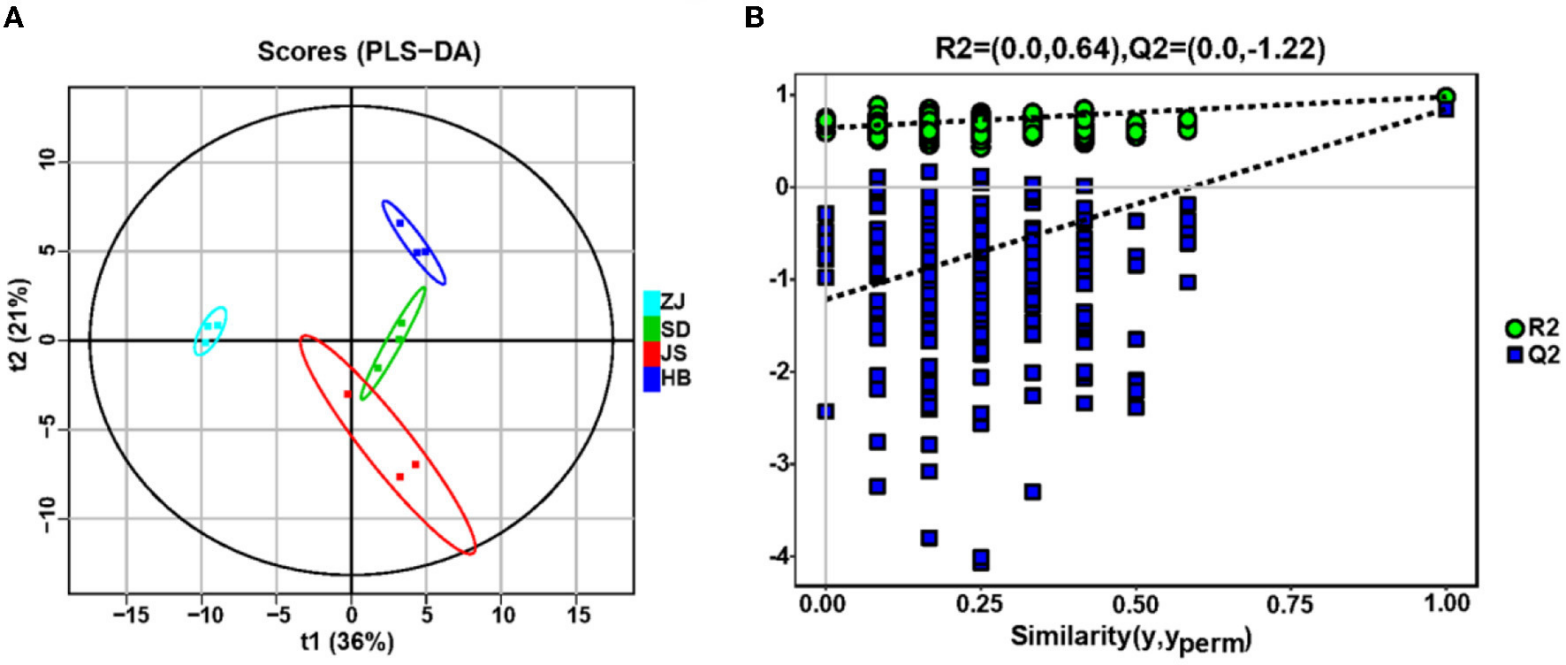
Metabolites of gut microbiota of *P. clarkii* from different habitats. **(A)** Partial least squares-discriminant analysis (PLS-DA). **(B)** Permutation test of PLS-DA for the fecal metabolomic profiles among the four groups.

GC–MS was used for understanding the metabolites of *P. clarkii* from four habitats. A total of 136 metabolites were exhibited, including 57 acids, 38 saccharides, 16 alcohols, 14 hydrocarbons, 3 ketones, 3 amines, 2 phenols, 2 esters, and 1 aldehyde (Supplementary Table S2). Through PLS-DA analysis (Figure 5A), the *P. clarkii* samples from four habitats can be effectively distinguished (R2X = 0.929, R2Y = 0.979, Q2Y = 0.847), and the model's capacity for modeling and prediction was strong. After the permutation test (Figure 5B), the model did not have overfitting, and the model verification was successfully confirmed because the sites where the Q2 regression line and the longitudinal axis intersected were below zero, indicating that these results can be used for the origin identification analysis of *P. clarkii*.

In the ZJ group, 63 compounds were identified, which was the least of all the groups. A total of 73 compounds were detected in SD, 70 in JS, and 72 in HB (Supplementary Table S2). There were obvious differences in compounds and total concentrations among different production areas. The compounds with the highest content were saccharides, followed by acids and alcohols. The HB group had much higher hydrocarbon and acid content than the other groups.

**Figure 6:**
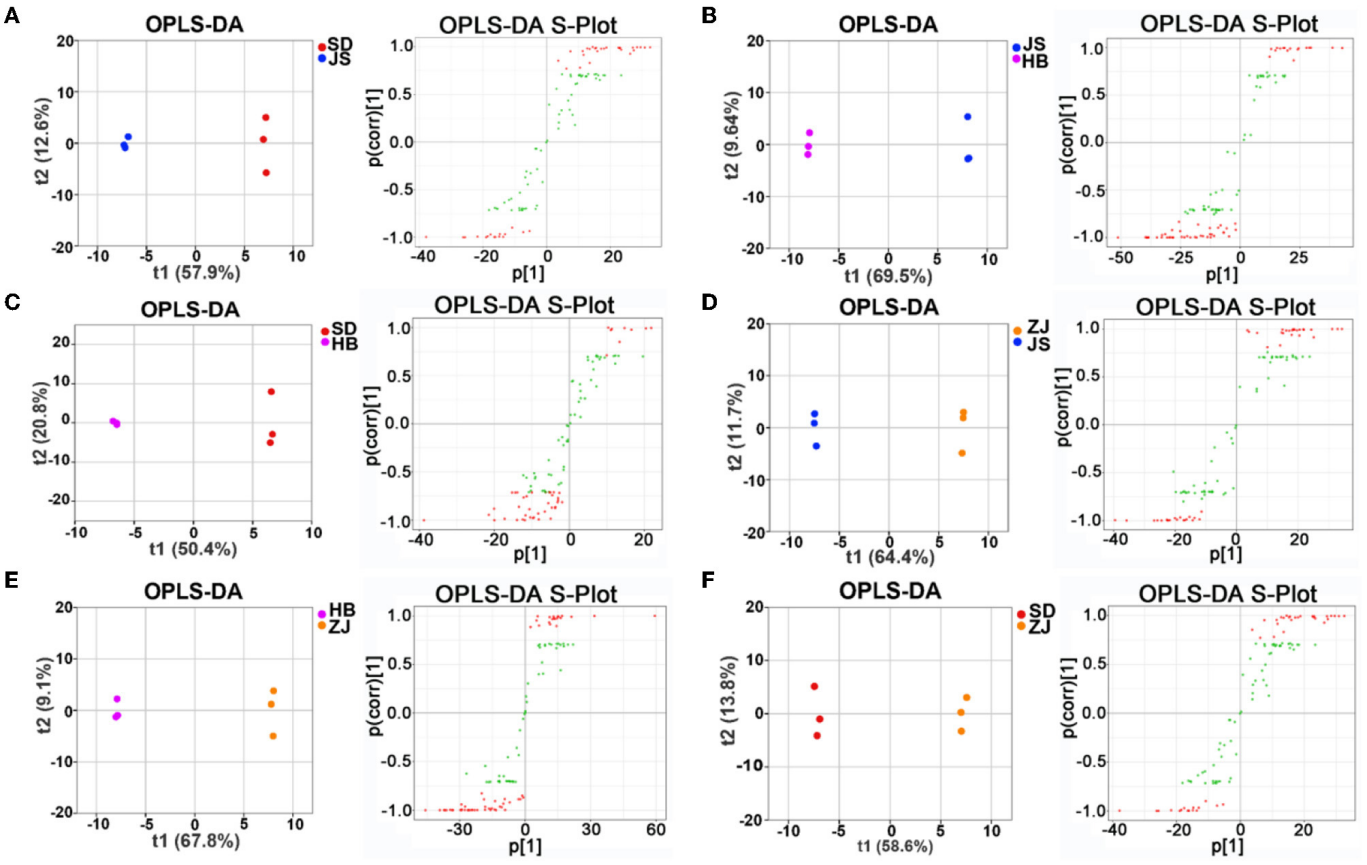
OPLS-DA assisted screening of differential metabolites between groups. **(A)** Screening of differential metabolites in the SD group compared with the JS group. **(B)** Screening of different metabolites in the JS group compared with the HB group. **(C)** Screening of different metabolites in the SD group compared with the HB group. **(D)** Screening of different metabolites in the ZJ group compared with the JS group. **(E)** Screening of different metabolites in the HB group compared with the ZJ group. **(F)** Screening of different metabolites in the SD group compared with the ZJ group.

Following that, orthogonal partial least squares-discriminant analysis was going on to examine biochemical indicators relative to distinct groups (VIP ≥ 1, *p* < 0.05) (Figures 6A–F). The contents of 43 distinct metabolites, including D-galactose, D-ribose, and L-isoleucine, were different between the SD group and the ZJ group. The 29 distinct metabolites, whose concentrations varied considerably between the SD and HB groups and included D-arabinose and L-hydroxyproline, were confirmed. There were 31 differential chemicals between the SD group and JS group, including D-galactose and α-linolenic acid. An overall of 54 distinct substances were detected in the HB group and ZJ group, the contents of which were significantly different, including DL-phenylalanine, D-arabinose, D-galactose, and L-isoleucine. There were 50 differential metabolites between the JS group and HB group, the contents of which were different, including 5-dodecenoic acid, D- (+)-trehalose, palatinose, and glycerol monostearate. There were 35 differential metabolites in the JS group and ZJ group, including D-galactose, palatinose, D- (+)-trehalose, decanoic acid, and butanedioic acid (Supplementary Tables S3–S7).

**Figure 7:**
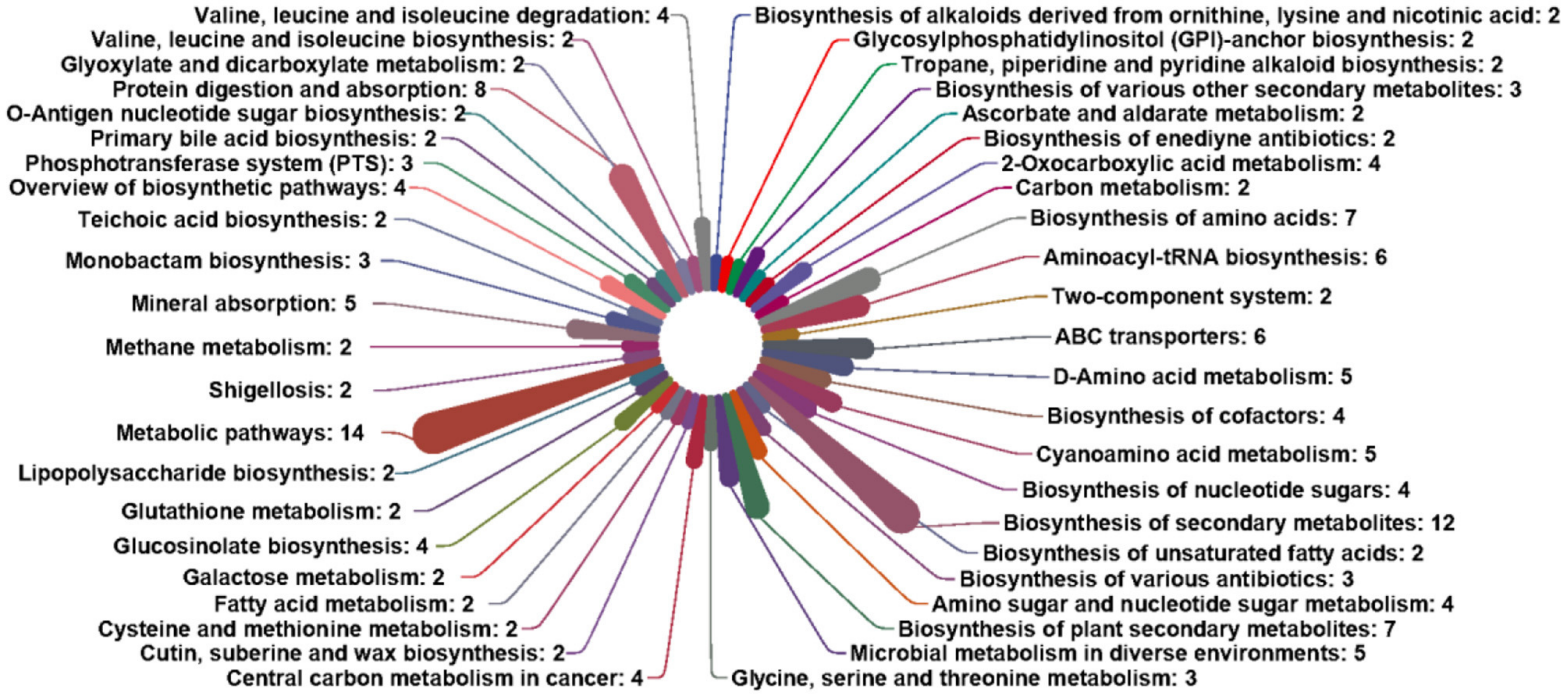
Differential metabolism pathway analysis.

Based on these metabolic markers, the pathways correlatively were determined in development (Figure 7). Only pathways with hits ≥ 2 were selected as potential target pathways. The third classification information of KEGG pathways included 14 metabolic markers: D-galactose, N-acetyl-D-glucosamine, D-arabinose, D-mannose, decanoic acid, L-alanine, L-isoleucine, glycine L-leucine, L-threonine, L-serine, L-valine, cholesterol, and cadaverine. These metabolic markers were involved in the Biosynthesis of secondary metabolites, metabolic pathways, and protein digestive absorption.

### Association analysis between differential species and differential metabolites

**Figure 8:**
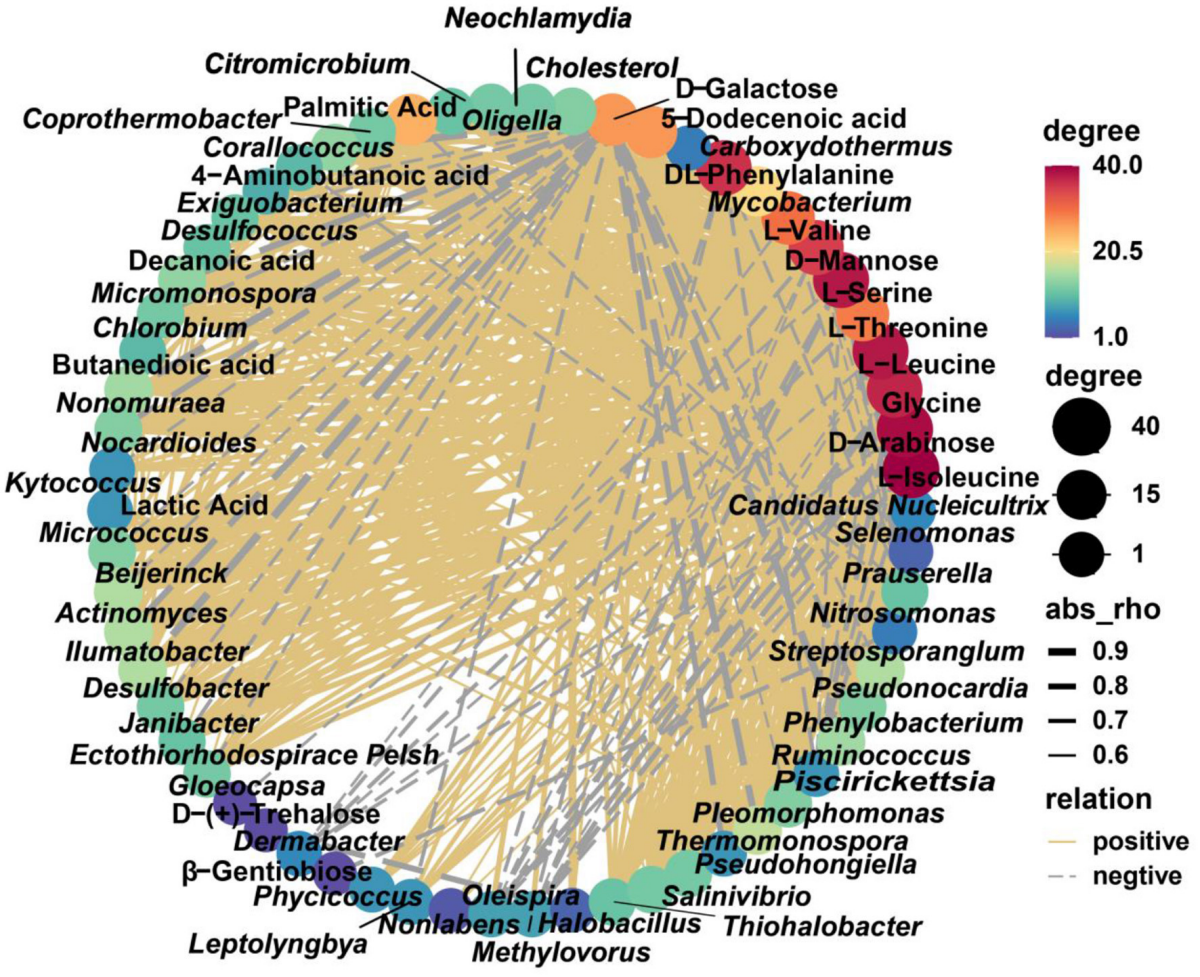
Network regulation diagram between differential species and differential metabolites (*p* < 0.05). Yellow solid line, positive correlation; gray dotted line, negative correlation.

Common differential metabolites with complete annotations were used for Spearman's correlation analysis with key differential genera, which revealed potential correlations between some microbe–metabolite pairs (*p* < 0.05). The element of differential metabolites in SD and ZJ was D-galactose, which was negatively correlated with *Thermomonospora, Kytococcus, Piscirickettsia, Ilumatobacter, Chlorobium*, and *Kytococcus*. The same situation occurred in the SD and JS groups. The differential metabolites between the HB group and ZJ group were attributed to D-arabinose and D-mannose, which were positively correlated with *Mycobacterium, Phycicoccus, Neochlamydia, Oligella, Thiohalobacter, Pseudohongiella, Exiguobacterium, Desulfococcus, Micromonospora, Nonomuraea, Nocardioides*, and *Actinomyces*, and were negatively correlated with *Oleispira, Methylovorus, Dermabacter*, and *Prauserella*. In JS compared to ZJ, β-gentiobiose was negatively correlated with *Nitrosomonas*. D-Galactose was negatively correlated with *Mycobacterium, Exiguobacterium, Thermomonospora, Micromonospora, Nonomuraea, Micrococcus, Piscirickettsia, Ilumatobacter, Streptosporangium*, and *Chlorobium*. The differential metabolites between the HB group and JS group were attributed to L-isoleucine, L-serine, L-threonine, L-leucine, glycine, and L-valine, which were positively correlated with *Mycobacterium, Phycicoccus, Neochlamydia, Janibacter, Oligella, Thiohalobacter, Pseudohongiella, Pleomorphomonas, Exiguobacterium, Desulfococcus, Micromonospora, Nonomuraea, Nocardioides, Nocardioides*, and *Actinomyces*, and were associated with *Dermabacter* negatively (Figure 8).

## Discussion

With the aquaculture continuous improvement, *P. clarkii* is a vital economic underpinning and forms an important component of the world's food supply (Yan et al., 2022). Studies discovered that survival temperature is a critical surrounding element. Each biont has a range of temperature tolerances and a suitable temperature for survival and growth (Araneda et al., 2020; Kir et al., 2023). The gut microbiota participates in many ways, covering immune gastrointestinal motility, regulation, and regulation of energetic metabolism (Xing et al., 2020). The multiplicity of the bacteria in aquatic animal products is greatly susceptible to temperature. For example, when the water temperature of cultured sea cucumbers is 14°C, no *Vibrio* can be detected in the intestinal tissue, but *Vibrio* is present at other temperatures, which testified that the culture temperature affected the quantity of *Vibrio* in the intestinal constituents of sea cucumbers (Gao et al., 2014). Temperature is also a principal element affecting the diversity of aquatic product microbiota. For example, on Day 80, the alpha diversity of the Chinese giant salamander decreased as the temperature rose. This pattern was, to some extent, inverse on Day 30 (Zhu et al., 2021). The test results manifested that *P. clarkii* had the highest diversity in the ZJ group, the most suitable temperature. Similarly, studies on *Litopenaeus vannamei* found that cold stress reduces the richness of microbial communities (Wang et al., 2020). The stability of the bacteria depends on the richness and diversity of the bacteria there. The discovery may indicate that *P. clarkii* reacts to temperature variations by changing the bacterial community as a whole to preserve homeostasis.

The gut microbiota of *P. clarkii* had three phyla with the greatest abundance, including Proteobacteria, Firmicutes, and Bacteroidetes (Xie et al., 2021). A similar result was obtained in this research. These were primary microorganisms in the bacteria of aquatic animals, such as *Larimichthys crocea* and *Eriocheir sinensis*, as an example (Gu et al., 2020; Ma et al., 2021). Numerous studies have indicated that Proteobacteria and Firmicutes play a vital part in the animals' adaptation process (Shin et al., 2015; Sun et al., 2022). Concerning this survey, a larger number of bacteria that responded to regional changes pertained to these phyla to a great extent. Scientific research has verified that the quantity of Firmicutes is favorably connected with material movement, but the amount of Bacteroidetes is directly correlated with nutrition metabolism (Turnbaugh et al., 2009). Bacteroidetes play a crucial role in promoting carbohydrate metabolism, while Firmicutes are responsible for promoting nutrient absorption. In this study, Proteobacteria and Bacteroidetes were more commonly found in ZJ. It showed that the ability of substance metabolism was improved and the growth and development speed were boosted in suitable temperature.

The gut microbiota's diversity and the structure of the bacterial community are linked to rising environmental temperatures, as indicated by the findings of the cluster analysis. The relative abundance of these genera generally increases and subsequently decreases as temperature rises, with certain groups exhibiting decreasing and then increasing. *Citrobacter* and *Enterobacter* were found in samples from different regions, but their abundance was greatly affected by temperature. Studies have found that *Enterobacter* contains several recognized pathogens and opportunistic bacteria associated with the occurrence of diseases in freshwater ornamental fish, such as goldfish (Walczak et al., 2017; Preena et al., 2021). *Citrobacter* infection, for instance, *Acipenser sinensis, Rhamdia quelen*, and zebrafish, has been linked to diseases that have been reported to occur (Lü et al., 2012; Junior et al., 2018; Yang et al., 2021). To date, infections by *Citrobacter* have already been verified and separated in some species, for instance, angelfish, catfish, and rainbow trout (Nawaz et al., 2008; Gallani et al., 2016; Duman et al., 2017). In general terms, *Citrobacter* is predominant in *P. clarkii* normally (Mullineaux-Sanders et al., 2019). Previous research on the gut microbiota of *P. clarkii* in different seasons found that the magnitude of *Citrobacter* in *P. clarkii* in autumn was more widespread than in summer (Liu et al., 2020). The average temperatures in June were 28.5°C in Hubei Province, 26.5°C in Jiangsu Province, 24.5°C in Zhejiang Province, and 17.5°C in Shandong Province. With rising temperatures, the study's findings revealed a tendency of initially declining and then increasing *Citrobacter* and *Enterobacter* numbers, with the JS group exhibiting the lowest abundance, indicating that temperature affects the amount of *Citrobacter* and *Enterobacter*. Regarding the study of the effect of temperature on the intestinal microbiota of salamander, *Citrobacter* showed the same trend change (Fontaine et al., 2018). In summary, this further confirmed the previous findings that under cold conditions settings, the infectivity of *Citrobacter* and *Enterobacter* may be increased. The same trend of change was also observed in *Aeromonas* in this investigation. *Aeromonas* made up a sizable amount of the bacteria in the HB group. *Aeromonas* are the main disease-producing bacteria in freshwater creatures (Qiu et al., 2022). *Aeromonas* infection of fish easily causes symptoms such as sepsis, enteritis, skin ulceration, and necrosis, such as *Pelodiscus sinensis* and *Betta splendens Regan* (Purivirojkul, 2012; Lv et al., 2021). Prior studies suggested that the number of *Aeromonas* and other bacteria in the gut of Chinese mitten crabs that were attacked by the white spot syndrome virus was significantly sick to shrimp (Cornejo-Granados et al., 2017). A new investigation on the susceptibility of crucian carp at different temperatures to *Aeromonas* infection found that golden carp at 33°C were more vulnerable (Jiang et al., 2020). The HB group had a high temperature, and the abundance of *Aeromonas* was also markedly higher than that in the other groups, showing that high temperature may be a considerable element affecting the abundance of *Aeromonas*. The findings indicated that a rise in harmful bacteria such as Aeromonas was mostly brought on by a rise in temperature. With rising temperatures, the quantity of *Mycobacterium* tended initially to grow and then decline, with the JS group having the maximum abundance. This outcome was in line with research on *Lithobates pipiens*, which discovered that the gut's *Mycobacterium* abundance increases with temperature. It is more frequent at 28°C than it is at 18°C (Kohl and Yahn, 2016).

A trend was also seen in some bacteria in response to temperature variations. With rising temperatures, there was a brief decline in *Planctomycetes*' abundance followed by a notable increase. Additionally, a warm climate was found to greatly enhance the quantity of *Planctomycetes* in the gut microbiome of tadpoles (Kohl and Yahn, 2016). With increased temperatures, there was a tendency for *Photobacterium* abundance to first decline and then increase. The normal growth of *Litopenaeus vannamei* was impacted by the invasion of *Photobacterium*, which was stimulated by an increase in *Photobacterium* when the temperature of the water rose (Al-Masqari et al., 2022). *Rhodococcus* abundance exhibited a trend of first growing and then declining as temperature rises. Temperature and the abundance of *Rhodococcu*s were directly correlated, with the exception of the HB group. Temperature rises had a substantial impact on the abundance of *Rhodococcus*, which in turn affects *Eriocheir sinensis*'s ability to grow normally (Guo et al., 2021). Animals' intestines and lungs could be infected with *Rhodococcus*, according to findings (Suzuki et al., 2021). Temperature and the abundance of *Vibrio* were inversely correlated. Temperature consistently affects the amount of *Vibrio* in the research of juvenile *Eriocheir sinensis* (Liang et al., 2023). According to some research, *Vibrio* is a harmful bacterium that can significantly affect growth and development and cause intestinal disorders (Fernández-Vélez et al., 2023; Samsing et al., 2023). In summary, temperature stress has a major impact on the makeup of the gut microbiota in *P. clarkii*.

The action of the microbiota was predicted, which supports a link between the microbiome community and metabolic action. The gut is more crucial for nutritional digestion and absorption and also for metabolism. In this study, using the KEGG database, we found that the gut microbiota of *P. clarkii* was mostly connected with metabolism or genetic information processing. Functional predictions indicated that cofactor, vitamin, amino acid, and nucleotide metabolism were affiliated with some pathways. The metabolism of energy and amino acids involves a sizable number of processes. These molecules play a critical role as mediators in the body's chemical conversion processes and the carbohydrate metabolism of all beings (Kolukisaoglu, 2020).

These consequences emphasize the potential influence of temperature on microbiota taxonomy and function. Metabolic analysis was then used to check the relative influence of the microbiota on the metabolites of *P. clarkii*. In this study, gut metabolites in *P. clarkii* from different regions were assessed, and metabolites that varied significantly between different temperatures were compared. The findings suggested a relationship between *P. clarkii's* gut microbiota and the host's ability to digest a source of nutrition. More specifically, we discovered a relationship between the relative abundance of several bacterial genera and the level of energy metabolism. It is noteworthy that intestinal polysaccharides are significantly broken down by bacteria belonging to the species *Bacteroides* (Ryan et al., 2020; McKee et al., 2021). The outcomes of the experiment demonstrated that, as temperature increased, the abundances of *Bacteroides* and N-acetyl-D-glucosamine initially decreased and subsequently increased. Intestinal homeostasis is significantly regulated by N-acetyl-D-glucosamine (Wang et al., 2022b). *Roseburia* possesses a complete genome and a core for the synthesis of amino acids. With rising temperatures, there was a pattern of first decreasing and then increasing abundances of glycine, L-leucine, L-serine, L-threonine, and L-valine. These were the highest in the HB group. *Roseburia* displayed similar trend features as well. *Pseudomonas* bacteria can create extracellular polysaccharides, which are mostly made of carbohydrates and aid in the formation of biofilms (Cavallero et al., 2020; Stoner et al., 2022). *Pseudomonas* and maltose abundances tended initially to rise and then fall as the temperature rose. Biofilm performs vital tasks such as energy conversion and material transportation. Carbohydrate metabolites can both store the energy substance starch and serve as the starting point for a variety of metabolic processes such as amino acid metabolism, citric acid cycle, and secondary metabolism (Stack and Gerlt, 2021). Mannose, fructose, and glucose can be transformed into three of them that play vital functions in secondary metabolism and amino acid metabolism. Amino acids directly influence many physiological pathways in living things and play a character controlling numerous metabolic processes, physical processes, and biochemical pathways of creatures (Chandel, 2021). To adjust to the changing temperature of the surroundings, a greater amount of energy must be used to maintain a stable condition. Thus, this offers a potential rationale for the connection between these bacteria's abundance and the materials' metabolism and energy absorption.

## Conclusion

This study preliminarily clarified the distinctions in gut microbiota and metabolites of *P. clarkii* at diverse temperatures from the four geographical zones. The present study showed that regional temperature had a greater effect on the gut microbiota of *P. clarkii*, especially some pathogenic microbiota. It was also found that temperature-induced changes in gut metabolites as well. It provides a basis for an in-depth investigation of the adaptation of *P. clarkii* to different geographical environments and provides a theoretical basis for the cultivation of *P. clarkii*.

## Data availability statement

The datasets presented in this study can be found in online repositories. The names of the repository/repositories and accession number(s) can be found in the article/Supplementary material.

## Ethics statement

The animal study was approved by the care and use of experimental animals complied with Ningbo University Laboratory Animal Center animal welfare laws, guidelines and policies as approved by no. SYXK (ZHE 2008-0110). The study was conducted in accordance with the local legislation and institutional requirements.

## Author contributions

SL: Writing – original draft, Data curation, Formal analysis, Resources. ZW: Formal analysis, Resources, Software, Validation, Writing – original draft. ZW: Data curation, Methodology, Visualization, Writing – original draft. QW: Formal analysis, Methodology, Resources, Writing – original draft. JZ: Investigation, Methodology, Project administration, Writing – original draft. RW: Project administration, Supervision, Writing – original draft. JH: Methodology, Project administration, Supervision, Writing – original draft, Writing – review & editing. XS: Funding acquisition, Resources, Supervision, Validation, Visualization, Writing – original draft, Writing – review & editing.
