# Supplementary material for: Comparison of the gut microbiota and metabolism in different regions of Red Swamp Crayfish (Procambarus clarkii)
Source: Front Microbiol. 2023 Dec 22;14:1289634. doi: 10.3389/fmicb.2023.1289634 (PMC10770849; doi:10.3389/fmicb.2023.1289634)
Supplement: Supplementary file 1 [file Table_1.docx]

**Table** **S1 The metabolites of *P. clarkii* from different origins**

| Component | Content (mg/g) | | | |  |  |
| --- | --- | --- | --- | --- | --- | --- |
|  | SD | JS | HB | ZJ | Pvalue | VIP |
| Acids |  |  |  |  |  |  |
| 1-monopalmitin | 39.86±39.97 | 38.01±33.77 | 86.41±4.26 | - | 0.025 | 0.494 |
| 2-butenedioic acid | 7.96±2.37 | - | 5.18±4.51 | 20.55±17.80 | 0.119 | 0.223 |
| 2-hydroxybutyric acid | - | - | 5.73±5.12 | - | 0.06 | 0.174 |
| 3 hydroxy-2,3-didehydrosebacic acid | 21.09±5.97 | - | 24.75±9.68 | 28.39±6.60 | 0.003 | 0.36 |
| 3-hydroxydodecanedioic acid | - | - | 17.31±15.66 | - | 0.063 | 0.295 |
| 3-hydroxydodecanoic acid | - | - | 47.46±41.21 | - | 0.053 | 0.499 |
| 3-trimethylsiloxypalmitic acid | 16.81±6.85 | - | 16.25±14.15 | - | 0.041 | 0.309 |
| 4-aminobutanoic acid | 101.83±32.59 | - | 5.58±2.22 | - | 0 | 1.176 |
| 5-dodecenoic acid | 122.91±44.14 | - | 265.61±3.95 | 29.72±11.43 | 0 | 1.015 |
| 9-decenoic acid | 1.11±1.93 | 21.79±19.39 | 43.74±7.07 | 33.11±18.93 | 0.03 | 0.616 |
| 9-octadecenoic acid | - | - | 6.95±1.12 | 18.09±5.56 | 0 | 0.255 |
| 9-tetradecenoic acid | - | - | 9.39±3.41 | - | 0 | 0.25 |
| Acetic acid | - | - | - | 43.78±17.43 | 0.001 | 0.345 |
| Alanylglycine | 28.35±26.62 | - | - | - | 0.074 | 0.55 |
| Benzenepentadecanoic acid | - | - | - | - | 1 | 0 |
| Butanedioic acid | 377.32±93.85 | 8.23±7.65 | 312.57±28.96 | 257.95±10.16 | 0 | 1.695 |
| DL-ornithine | 14.80±16.59 | - | - | - | 0.145 | 0.384 |
| DL-phenylalanine | 197.30±59.42 | 43.35±42.00 | 365.84±7.42 | - | 0 | 1.128 |
| Decanoic acid | 291.19±86.90 | 4.14±3.59 | 301.19±22.57 | 79.04±1.04 | 0 | 1.398 |
| Erythro-pentonic acid | - | - | 24.42±33.56 | - | 0.267 | 0.32 |
| Dodecanoic acid | 19.46±8.36 | - | 77.69±3.74 | - | 0 | 0.596 |
| Glycine | 291.28±171.76 | 99.51±39.88 | 580.76±82.76 | 12.18±12.60 | 0 | 1.364 |
| Hexadecanoic acid | - | - | - | 19.02±11.76 | 0.009 | 0.219 |
| Hexanoic acid | - | 21.00±0.70 | 5.89±5.26 | 6.09±5.95 | 0.001 | 0.413 |
| Lactic acid | 2169.58±590.70 | 1153.93±85.77 | 2252.60±109.38 | 1995.63±68.15 | 0.008 | 2.48 |
| L-alanine | - | 482.88±258.85 | - | - | 0.004 | 1.815 |
| L-aspartic acid | 63.01±24.06 | 14.73±12.76 | - | - | 0.001 | 0.851 |
| L-hydroxyproline | - | - | 55.37±4.70 | - | 0 | 0.614 |
| L-isoleucine | 348.60±107.93 | 65.69±57.28 | 570.54±20.55 | - | 0 | 1.436 |
| L-leucine | 381.61±124.25 | 183.84±103.16 | 620.89±38.60 | 78.24±104.17 | 0.001 | 1.307 |
| L-methionine | 147.52±48.94 | 24.33±14.46 | 140.31±53.09 | - | 0.002 | 0.897 |
| L-proline | - | 20.20±6.21 | - | - | 0 | 0.37 |
| L-serine | 197.06±9.46 | 177.01±42.53 | 474.15±61.81 | 47.66±50.46 | 0 | 1.293 |
| L-threonine | 232.86±192.59 | 244.96±62.63 | 542.43±94.61 | 133.28±58.12 | 0.012 | 1.306 |
| L-valine | 282.81±80.65 | 182.77±95.46 | 531.46±20.06 | 153.61±88.45 | 0.001 | 1.197 |
| Malic acid | 37.70±6.56 | 5.76±5.03 | 52.35±5.21 | 37.99±4.00 | 0 | 0.453 |
| Mandelic acid | - | 3.11±2.70 | - | 13.51±4.34 | 0.001 | 0.199 |
| N, N-dimethylglycine | - | 34.08±14.43 | - | - | 0.001 | 0.488 |
| Nonanoic acid | - | - | 3.24±2.83 | - | 0.053 | 0.13 |
| N-α-acetyl-L-lysine | 215.66±219.56 | - | - | - | 0.102 | 1.553 |
| Octanoic acid | - | - | - | 17.90±3.66 | 0 | 0.226 |
| Oleic acid | 8.60±8.02 | - | 68.23±10.38 | - | 0 | 0.611 |
| Palmitic acid | 201.85±76.26 | - | 246.87±16.77 | - | 0 | 1.135 |
| Pentanedioic acid | 44.05±23.25 | - | 108.31±18.78 | - | 0 | 0.648 |
| Phosphoric acid | - | - | - | 45.39±42.12 | 0.07 | 0.315 |
| Phthalic acid | - | - | - | 11.28±5.86 | 0.003 | 0.172 |
| Pipecolic acid | - | - | - | 78.27±99.06 | 0.212 | 0.38 |
| Propanedioic acid | 39.79±14.59 | - | 56.13±8.32 | 120.36±16.84 | 0 | 0.593 |
| Propanoic acid | 33.37±10.75 | 12.13±11.42 | 20.06±17.49 | 37.40±2.98 | 0.099 | 0.386 |
| Pyroglutamic acid | 95.56±97.14 | 35.34±31.31 | 232.79±75.69 | 239.80±9.37 | 0.01 | 0.95 |
| Sulfurous acid | - | 13.21±2.45 | - | - | 0 | 0.302 |
| Stearic acid | 14.87±15.61 | - | 65.41±14.55 | - | 0 | 0.543 |
| Tridecanoic acid | - | - | - | 10.36±5.92 | 0.006 | 0.163 |
| Tyrosine | 84.19±32.52 | - | 142.38±132.81 | - | 0.089 | 0.672 |
| Undecanoic acid | 51.85±45.03 | - | 50.20±36.11 | 18.15±15.80 | 0.172 | 0.506 |
| Valproic acid | - | - | 41.26±35.79 | - | 0.052 | 0.493 |
| α-linolenic acid | 10.19±1.05 | - | - | 40.98±55.82 | 0.299 | 0.28 |
| Saccharides |  |  |  |  |  |  |
| 2-α-mannobiose | 7.41±6.60 | 37.15±35.77 | - | 5.88±5.84 | 0.138 | 0.429 |
| 3-α-mannobiose | 6.73±7.81 | 42.44±36.64 | - | 22.29±1.37 | 0.093 | 0.442 |
| Arabinofuranose | - | 4.25±3.99 | - | - | 0.074 | 0.166 |
| D-(-)-lyxose | - | 8.58±3.77 | - | - | 0.001 | 0.241 |
| D-(-)-ribofuranose | 4.41±4.66 | - | - | 14.55±5.92 | 0.004 | 0.213 |
| D-(+)-cellobiose | 30.37±38.09 | 8.08±8.19 | - | - | 0.258 | 0.481 |
| D-(+)-talofuranose | - | 13.97±2.57 | - | - | 0 | 0.317 |
| D-(+)-talose | 1214.13±1054.38 | 2150.31±1870.94 | 2070.16±2651.33 | 2326.86±70.10 | 0.857 | 2.498 |
| D-(+)-trehalose | 79.13±25.56 | 248.03±5.70 | - | - | 0 | 1.085 |
| D-(+)-turanose | 7.49±7.20 | 3.66±4.78 | - | - | 0.184 | 0.232 |
| D-(+)-xylose | - | - | - | 156.05±135.21 | 0.052 | 0.595 |
| D-allose | 2895.42±4160.51 | 5851.58±7563.27 | 1974.55±2442.21 | 211.93±12.79 | 0.514 | 4.042 |
| D-arabinose | 80.17±24.71 | 12.54±4.17 | 406.74±20.70 | - | 0 | 1.419 |
| D-fructose | 10.46±10.06 | 3.31±3.24 | - | 35.02±30.68 | 0.105 | 0.289 |
| D-galactose | - | 67.65±4.51 | - | 11014.05±208.71 | 0 | 5.659 |
| D-glucose | 3819.88±3615.97 | 4471.69±7705.08 | 2069.40±2601.48 | 4.73±4.10 | 0.629 | 3.836 |
| D-lactose | - | 30.78±26.70 | - | - | 0.052 | 0.419 |
| D-lyxose | - | 5.37±4.99 | - | - | 0.07 | 0.17 |
| D-mannose | 180.97±156.74 | 24.65±21.46 | 431.84±80.20 | - | 0.001 | 1.234 |
| D-psicose | - | - | - | 56.11±48.64 | 0.052 | 0.358 |
| D-ribose | - | - | - | 195.57±20.52 | 0 | 0.753 |
| Galactofuranose | 10.38±9.61 | - | 17.53±15.30 | - | 0.116 | 0.234 |
| Glyceryl-glycoside | 12.45±3.42 | - | - | - | 0 | 0.422 |
| Levoglucosan | - | 9.08±8.45 | - | - | 0.071 | 0.232 |
| L-rhamnose | 48.84±60.93 | 7.38±6.73 | 16.95±10.23 | - | 0.302 | 0.543 |
| Maltose | 11.39±4.78 | 17.28±0.42 | 8.02±6.94 | 48.14±42.66 | 0.173 | 0.268 |
| Methyl galactoside | 22.30±5.23 | 52.35±6.99 | - | - | 0 | 0.478 |
| Methyl α-D-ribofuranoside | - | 3.99±3.50 | - | - | 0.055 | 0.151 |
| N-acetyl-D-galactosamine | - | - | 15.02±13.18 | 12.59±11.22 | 0.126 | 0.28 |
| N-acetyl-D-glucosamine | 964.25±281.31 | 274.06±11.93 | 1623.11±155.20 | 600.12±520.54 | 0.004 | 2.182 |
| Palatinose | - | 61.61±1.82 | - | - | 0 | 0.662 |
| Sedoheptulose | - | 6.64±4.40 | - | - | 0.013 | 0.207 |
| Sucrose | - | 1.48±1.29 | - | - | 0.052 | 0.099 |
| β-D-galactopyranoside | 60.15±58.22 | - | 8.46±7.79 | - | 0.101 | 0.784 |
| β-D-glucopyranose | 12.10±4.54 | - | - | - | 0 | 0.412 |
| β-L-galactopyranoside | - | - | - | 9.32±8.10 | 0.053 | 0.145 |
| β-L-mannofuranose | - | - | - | 13.98±12.22 | 0.054 | 0.178 |
| β-gentiobiose | 54.43±77.20 | 259.11±30.29 | 9.09±5.16 | 8.25±0.63 | 0 | 1.138 |
| Alcohols |  |  |  |  |  |  |
| 1,2,4-butanetriol | - | - | - | 9.87±2.08 | 0 | 0.168 |
| 1,3-dioxolane-2-methanol | - | 8.87±1.26 | - | - | 0 | 0.252 |
| 2-tridecanol | 26.24±27.19 | - | - | - | 0.109 | 0.518 |
| 7-tetradecen-1-ol | - | - | - | 17.46±15.18 | 0.053 | 0.199 |
| Campesterol | - | - | 19.22±2.41 | - | 0 | 0.36 |
| Cholest-5-en-3-ol | - | - | - | 21.60±10.24 | 0.002 | 0.239 |
| Cholesterol | 7.63±6.73 | - | 263.68±54.57 | 24.04±8.13 | 0 | 1.308 |
| D-mannitol | - | - | - | 29.18±25.48 | 0.054 | 0.258 |
| D-pinitol | - | 19.01±12.70 | - | - | 0.014 | 0.334 |
| Muco-inositol | - | 4.93±4.37 | - | - | 0.058 | 0.167 |
| Myo-inositol | 38.21±33.09 | 42.48±1.60 | 46.29±5.38 | - | 0.032 | 0.325 |
| Ribitol | - | 31.55±49.28 | - | - | 0.361 | 0.423 |
| Scyllo-inositol | 29.51±25.59 | 11.64±0.70 | 15.69±2.20 | 9.98±1.49 | 0.305 | 0.368 |
| Tromethamine | - | 10.96±2.80 | - | 12.72±2.35 | 0 | 0.271 |
| Xylitol | - | - | 60.14±54.75 | - | 0.065 | 0.564 |
| Isoborneol | - | - | 1493.52±104.34 | - | 0 | 3.177 |
| Hydrocardns |  |  |  |  |  |  |
| Benzene | 9.59±13.86 | - | - | - | 0.303 | 0.301 |
| Pyrazine | - | - | 13.26±0.63 | - | 0 | 0.299 |
| Decane | 4.07±3.81 | - | - | 16.77±3.12 | 0 | 0.226 |
| Disilathiane | - | 40.02±45.32 | - | - | 0.15 | 0.444 |
| Dodecaneac | - | 11.64±7.03 | 6.56±5.84 | - | 0.038 | 0.298 |
| Eicosanea | - | 4.27±3.99 | - | - | 0.072 | 0.154 |
| Heptacosane | 59.95±95.52 | 6.36±1.60 | - | - | 0.4 | 0.664 |
| Heptasiloxane | - | 3.59±3.23 | 14.50±9.00 | 15.84±7.40 | 0.029 | 0.321 |
| L-5-oxoproline | 155.69±64.27 | - | 239.65±43.65 | - | 0 | 0.998 |
| Pentasiloxane | 27.36±9.68 | 31.91±1.08 | 41.67±3.88 | 59.84±7.55 | 0.001 | 0.355 |
| Propane | - | - | - | 5.42±4.92 | 0.064 | 0.111 |
| Tetrasiloxane | 11.32±9.83 | 17.07±4.64 | 20.46±1.16 | 47.28±2.09 | 0 | 0.338 |
| Trisiloxane | 35.32±31.70 | - | - | 12.34±4.37 | 0.081 | 0.63 |
| Ketones |  |  |  |  |  |  |
| 1-propanone | - | - | - | 9.72±9.29 | 0.08 | 0.144 |
| 2-pyrrolidinone | 16.34±14.39 | 32.11±6.37 | 85.05±15.07 | - | 0 | 0.696 |
| Amines |  |  |  |  |  |  |
| Amphetamine | 68.98±60.97 | 28.65±6.81 | 134.50±7.07 | - | 0.004 | 0.631 |
| Putrescine | 9.15±3.21 | - | 27.94±5.73 | - | 0 | 0.341 |
| Phenols |  |  |  |  |  |  |
| 2,6-bis(tert-butyl)phenol | 20.60±3.90 | 4.89±4.28 | 18.09±2.21 | 22.22±19.25 | 0.22 | 0.274 |
| Phenol | - | 28.36±25.72 | 18.91±17.97 | - | 0.138 | 0.514 |
| Esters |  |  |  |  |  |  |
| 2-palmitoylglycerol | 7.07±6.68 | 7.73±1.17 | 13.94±5.27 | 9.81±8.51 | 0.534 | 0.164 |
| Glycerol monostearate | 43.64±16.64 | - | 54.26±1.94 | 3.62±3.32 | 0 | 0.524 |
| Aldehydes |  |  |  |  |  |  |
| Propanal | - | - | 10.80±4.05 | - | 0 | 0.268 |
