# Supplementary material for: Comparison of the gut microbiota and metabolism in different regions of Red Swamp Crayfish (Procambarus clarkii)
Source: Front Microbiol. 2023 Dec 22;14:1289634. doi: 10.3389/fmicb.2023.1289634 (PMC10770849; doi:10.3389/fmicb.2023.1289634)
Supplement: Supplementary file 2 [file Table_2.docx]

**Table S2 The difference in metabolites between the SD group and JS group.**

| Name | VIP | p_value | FDR | Type |
| --- | --- | --- | --- | --- |
| Palatinose | 1.33 | 0.00 | 0.02 | up |
| D-galactose | 1.33 | 0.00 | 0.05 | up |
| Hexanoic acid | 1.33 | 0.00 | 0.02 | up |
| α-linolenic acid | 1.32 | 0.00 | 0.07 | down |
| D-(+)-trehalose | 1.32 | 0.01 | 0.08 | up |
| 1,3-dioxolane-2-methanol | 1.31 | 0.01 | 0.08 | up |
| D-(+)-talofuranose | 1.31 | 0.01 | 0.10 | up |
| Sulfurous acid | 1.30 | 0.01 | 0.10 | up |
| Malic acid | 1.28 | 0.00 | 0.07 | down |
| Tromethamine | 1.28 | 0.02 | 0.15 | up |
| Butanedioic acid | 1.28 | 0.02 | 0.15 | down |
| 3 hydroxy-2,3-didehydrosebacic acid | 1.26 | 0.03 | 0.15 | down |
| Methyl galactoside | 1.26 | 0.01 | 0.08 | up |
| Glyceryl-glycoside | 1.26 | 0.02 | 0.15 | down |
| Decanoic acid | 1.26 | 0.03 | 0.15 | down |
| 4-aminobutanoic acid | 1.25 | 0.03 | 0.15 | down |
| 2-butenedioic acid | 1.24 | 0.03 | 0.15 | down |
| L-proline | 1.24 | 0.03 | 0.15 | up |
| 2,6-bis(tert-butyl)phenol | 1.24 | 0.01 | 0.10 | down |
| 5-dodecenoic acid | 1.23 | 0.04 | 0.15 | down |
| Propanedioic acid | 1.23 | 0.04 | 0.15 | down |
| D-arabinose | 1.23 | 0.04 | 0.15 | down |
| Palmitic acid | 1.22 | 0.04 | 0.15 | down |
| Glycerol monostearate | 1.22 | 0.05 | 0.15 | down |
| Putrescine | 1.22 | 0.04 | 0.15 | down |
| Tyrosine | 1.22 | 0.05 | 0.15 | down |
| β-gentiobiose | 1.21 | 0.03 | 0.15 | up |
| L-methionine | 1.21 | 0.04 | 0.15 | down |
| β-D-glucopyranose | 1.20 | 0.04 | 0.15 | down |
| L-isoleucine | 1.19 | 0.03 | 0.15 | down |
| DL-phenylalanine | 1.18 | 0.03 | 0.15 | down |
