# Supplementary material for: Comparison of the gut microbiota and metabolism in different regions of Red Swamp Crayfish (Procambarus clarkii)
Source: Front Microbiol. 2023 Dec 22;14:1289634. doi: 10.3389/fmicb.2023.1289634 (PMC10770849; doi:10.3389/fmicb.2023.1289634)
Supplement: Supplementary file 3 [file Table_3.docx]

**Table S3 The difference in metabolites between the JS group and HB group.**

| Name | VIP | p_value | FDR | Type |
| --- | --- | --- | --- | --- |
| 5-dodecenoic acid | 1.20 | 0.00 | 0.00 | up |
| D-(+)-trehalose | 1.20 | 0.00 | 0.01 | down |
| Palatinose | 1.20 | 0.00 | 0.01 | down |
| Glycerol monostearate | 1.20 | 0.00 | 0.01 | up |
| Dodecanoic acid | 1.20 | 0.00 | 0.01 | up |
| Pyrazine | 1.20 | 0.00 | 0.01 | up |
| D-arabinose | 1.20 | 0.00 | 0.01 | up |
| D-galactose | 1.20 | 0.00 | 0.01 | down |
| Palmitic acid | 1.20 | 0.00 | 0.01 | up |
| Isoborneol | 1.20 | 0.00 | 0.01 | up |
| Decanoic acid | 1.20 | 0.00 | 0.01 | up |
| L-hydroxyproline | 1.20 | 0.00 | 0.01 | up |
| Amphetamine | 1.20 | 0.00 | 0.00 | up |
| Butanedioic acid | 1.20 | 0.00 | 0.01 | up |
| N-acetyl-D-glucosamine | 1.19 | 0.00 | 0.02 | up |
| L-isoleucine | 1.19 | 0.00 | 0.01 | up |
| β-gentiobiose | 1.19 | 0.00 | 0.02 | down |
| Lactic acid | 1.19 | 0.00 | 0.01 | insig |
| DL-phenylalanine | 1.19 | 0.00 | 0.02 | up |
| Campesterol | 1.19 | 0.01 | 0.02 | up |
| Methyl galactoside | 1.19 | 0.01 | 0.03 | down |
| Malic acid | 1.19 | 0.00 | 0.01 | up |
| Propanedioic acid | 1.19 | 0.01 | 0.03 | up |
| Oleic acid | 1.19 | 0.01 | 0.03 | up |
| Pentanedioic acid | 1.18 | 0.01 | 0.03 | up |
| L-5-oxoproline | 1.18 | 0.01 | 0.03 | up |
| 9-octadecenoic acid | 1.18 | 0.01 | 0.03 | up |
| Glycine | 1.18 | 0.00 | 0.02 | up |
| D-(+)-talofuranose | 1.17 | 0.01 | 0.03 | down |
| Sulfurous acid | 1.17 | 0.01 | 0.03 | down |
| D-mannose | 1.17 | 0.01 | 0.03 | up |
| Cholesterol | 1.17 | 0.01 | 0.04 | up |
| Putrescine | 1.17 | 0.01 | 0.04 | up |
| Stearic acid | 1.16 | 0.02 | 0.04 | up |
| L-leucine | 1.16 | 0.01 | 0.03 | up |
| L-serine | 1.16 | 0.00 | 0.02 | up |
| Tromethamine | 1.15 | 0.02 | 0.05 | down |
| L-valine | 1.15 | 0.02 | 0.05 | up |
| Heptacosane | 1.14 | 0.02 | 0.05 | down |
| 2-pyrrolidinone | 1.13 | 0.01 | 0.04 | up |
| Hexanoic acid | 1.13 | 0.04 | 0.08 | down |
| L-proline | 1.13 | 0.03 | 0.07 | down |
| 2,6-bis(tert-butyl)phenol | 1.12 | 0.02 | 0.05 | up |
| L-threonine | 1.10 | 0.01 | 0.04 | up |
| 3 hydroxy-2,3-didehydrosebacic acid | 1.09 | 0.05 | 0.10 | up |
| 9-tetradecenoic acid | 1.09 | 0.04 | 0.09 | up |
| Propanal | 1.09 | 0.04 | 0.09 | up |
| Pyroglutamic acid | 1.09 | 0.03 | 0.07 | up |
| Pentasiloxane | 1.09 | 0.04 | 0.09 | insig |
| 4-aminobutanoic acid | 1.05 | 0.05 | 0.10 | up |
