# Supplementary material for: Comparison of the gut microbiota and metabolism in different regions of Red Swamp Crayfish (Procambarus clarkii)
Source: Front Microbiol. 2023 Dec 22;14:1289634. doi: 10.3389/fmicb.2023.1289634 (PMC10770849; doi:10.3389/fmicb.2023.1289634)
Supplement: Supplementary file 4 [file Table_4.docx]

**Table S4 The difference in metabolites between the SD group and HB group.**

| Name | VIP | p_value | FDR | Type |
| --- | --- | --- | --- | --- |
| Pyrazine | 1.39 | 0.00 | 0.04 | up |
| Isoborneol | 1.39 | 0.00 | 0.04 | up |
| D-arabinose | 1.38 | 0.00 | 0.01 | up |
| L-hydroxyproline | 1.38 | 0.00 | 0.04 | up |
| α-linolenic acid | 1.38 | 0.00 | 0.04 | down |
| Campesterol | 1.37 | 0.01 | 0.05 | up |
| Dodecanoic acid | 1.37 | 0.00 | 0.04 | up |
| 9-decenoic acid | 1.37 | 0.01 | 0.05 | up |
| 9-octadecenoic acid | 1.36 | 0.01 | 0.07 | up |
| L-serine | 1.35 | 0.01 | 0.09 | up |
| Oleic acid | 1.35 | 0.00 | 0.04 | up |
| Cholesterol | 1.35 | 0.01 | 0.09 | up |
| Methyl galactoside | 1.34 | 0.02 | 0.09 | down |
| 2-pyrrolidinone | 1.32 | 0.00 | 0.05 | up |
| 5-dodecenoic acid | 1.31 | 0.03 | 0.13 | up |
| Glyceryl-glycoside | 1.31 | 0.02 | 0.11 | down |
| D-(+)-trehalose | 1.30 | 0.03 | 0.13 | down |
| L-valine | 1.30 | 0.03 | 0.12 | insig |
| 4-aminobutanoic acid | 1.30 | 0.04 | 0.13 | down |
| Putrescine | 1.28 | 0.01 | 0.09 | up |
| DL-phenylalanine | 1.28 | 0.04 | 0.13 | insig |
| L-aspartic acid | 1.27 | 0.05 | 0.14 | down |
| Stearic acid | 1.26 | 0.01 | 0.09 | up |
| β-D-glucopyranose | 1.25 | 0.04 | 0.14 | down |
| 9-tetradecenoic acid | 1.24 | 0.04 | 0.14 | up |
| Propanal | 1.23 | 0.04 | 0.14 | up |
| Pentanedioic acid | 1.22 | 0.02 | 0.11 | up |
| N-acetyl-D-glucosamine | 1.21 | 0.04 | 0.13 | insig |
| Malic acid | 1.17 | 0.04 | 0.14 | insig |
