# Supplementary material for: Comparison of the gut microbiota and metabolism in different regions of Red Swamp Crayfish (Procambarus clarkii)
Source: Front Microbiol. 2023 Dec 22;14:1289634. doi: 10.3389/fmicb.2023.1289634 (PMC10770849; doi:10.3389/fmicb.2023.1289634)
Supplement: Supplementary file 5 [file Table_5.docx]

**Table S5 The difference in metabolites between the ZJ group and JS group.**

| Name | VIP | p_value | FDR | Type |
| --- | --- | --- | --- | --- |
| D-galactose | 1.27 | 0.00 | 0.01 | down |
| Palatinose | 1.27 | 0.00 | 0.01 | up |
| D-(+)-trehalose | 1.27 | 0.00 | 0.01 | up |
| Myo-inositol | 1.27 | 0.00 | 0.01 | up |
| Decanoic acid | 1.27 | 0.00 | 0.01 | down |
| Butanedioic acid | 1.27 | 0.00 | 0.00 | down |
| D-ribose | 1.26 | 0.00 | 0.04 | down |
| β-gentiobiose | 1.26 | 0.00 | 0.04 | up |
| Lactic acid | 1.26 | 0.00 | 0.01 | insig |
| Methyl galactoside | 1.26 | 0.01 | 0.05 | up |
| Propanedioic acid | 1.25 | 0.01 | 0.05 | down |
| 1,3-dioxolane-2-methanol | 1.25 | 0.01 | 0.05 | up |
| Pyroglutamic acid | 1.25 | 0.00 | 0.04 | down |
| Tetrasiloxane | 1.25 | 0.00 | 0.03 | down |
| Malic acid | 1.24 | 0.00 | 0.02 | down |
| Decane | 1.24 | 0.01 | 0.06 | down |
| Sulfurous acid | 1.24 | 0.01 | 0.06 | up |
| D-(+)-talofuranose | 1.24 | 0.01 | 0.06 | up |
| 2-pyrrolidinone | 1.24 | 0.01 | 0.07 | up |
| Octanoic acid | 1.23 | 0.01 | 0.07 | down |
| 1,2,4-butanetriol | 1.23 | 0.01 | 0.07 | down |
| 3-hydroxy-2,3-didehydrosebacic acid | 1.23 | 0.02 | 0.08 | down |
| Amphetamine | 1.23 | 0.02 | 0.08 | up |
| Pentasiloxane | 1.21 | 0.02 | 0.09 | insig |
| Heptacosane | 1.20 | 0.02 | 0.09 | up |
| L-proline | 1.19 | 0.03 | 0.11 | up |
| 9-octadecenoic acid | 1.19 | 0.03 | 0.11 | down |
| Cholesterol | 1.18 | 0.04 | 0.12 | down |
| D-arabinose | 1.17 | 0.04 | 0.12 | up |
| Hexanoic acid | 1.16 | 0.05 | 0.14 | up |
| Trisiloxane | 1.16 | 0.04 | 0.13 | down |
| 5-dodecenoic acid | 1.16 | 0.05 | 0.14 | down |
| Acetic acid | 1.15 | 0.05 | 0.14 | down |
| Mandelic acid | 1.11 | 0.03 | 0.12 | down |
| L-serine | 1.10 | 0.03 | 0.11 | up |
