# Supplementary material for: Comparison of the gut microbiota and metabolism in different regions of Red Swamp Crayfish (Procambarus clarkii)
Source: Front Microbiol. 2023 Dec 22;14:1289634. doi: 10.3389/fmicb.2023.1289634 (PMC10770849; doi:10.3389/fmicb.2023.1289634)
Supplement: Supplementary file 6 [file Table_6.docx]

**Table S6 The difference of metabolites between the HB group and ZJ group.**

| Name | VIP | p_value | FDR | Type |
| --- | --- | --- | --- | --- |
| DL-phenylalanine | 1.23 | 0.00 | 0.00 | down |
| D-galactose | 1.23 | 0.00 | 0.00 | up |
| L-isoleucine | 1.23 | 0.00 | 0.01 | down |
| 1-monopalmitin | 1.23 | 0.00 | 0.01 | down |
| D-arabinose | 1.23 | 0.00 | 0.01 | down |
| Dodecanoic acid | 1.23 | 0.00 | 0.01 | down |
| 5-dodecenoic acid | 1.23 | 0.00 | 0.00 | down |
| Glycerol monostearate | 1.23 | 0.00 | 0.00 | down |
| Amphetamine | 1.23 | 0.00 | 0.01 | down |
| Pyrazine | 1.23 | 0.00 | 0.01 | down |
| Isoborneol | 1.23 | 0.00 | 0.01 | down |
| Palmitic acid | 1.23 | 0.00 | 0.01 | down |
| Tetrasiloxane | 1.23 | 0.00 | 0.00 | up |
| 3-α-mannobiose | 1.23 | 0.00 | 0.01 | up |
| L-hydroxyproline | 1.23 | 0.00 | 0.02 | down |
| Decanoic acid | 1.23 | 0.00 | 0.02 | down |
| D-ribose | 1.22 | 0.00 | 0.02 | up |
| Myo-inositol | 1.22 | 0.00 | 0.02 | down |
| Campesterol | 1.22 | 0.01 | 0.03 | down |
| Glycine | 1.22 | 0.01 | 0.03 | down |
| Oleic acid | 1.21 | 0.01 | 0.03 | down |
| Pentanedioic acid | 1.21 | 0.01 | 0.04 | down |
| 2-pyrrolidinone | 1.21 | 0.01 | 0.04 | down |
| L-5-oxoproline | 1.21 | 0.01 | 0.04 | down |
| L-serine | 1.21 | 0.00 | 0.01 | down |
| D-mannose | 1.21 | 0.01 | 0.04 | down |
| L-leucine | 1.20 | 0.01 | 0.03 | down |
| Decane | 1.20 | 0.01 | 0.04 | up |
| Tromethamine | 1.20 | 0.01 | 0.04 | up |
| Putrescine | 1.20 | 0.01 | 0.04 | down |
| Stearic acid | 1.19 | 0.02 | 0.04 | down |
| Cholesterol | 1.19 | 0.02 | 0.04 | down |
| Octanoic acid | 1.19 | 0.01 | 0.04 | up |
| L-valine | 1.19 | 0.01 | 0.04 | down |
| L-threonine | 1.18 | 0.01 | 0.03 | down |
| 1,2,4-butanetriol | 1.18 | 0.01 | 0.04 | up |
| Propanedioic acid | 1.17 | 0.01 | 0.04 | up |
| Mandelic acid | 1.13 | 0.03 | 0.08 | up |
| L-methionine | 1.13 | 0.04 | 0.10 | down |
| Acetic acid | 1.11 | 0.05 | 0.10 | up |
| Trisiloxane | 1.11 | 0.04 | 0.09 | up |
| 9-tetradecenoic acid | 1.10 | 0.04 | 0.09 | down |
| Propanal | 1.10 | 0.04 | 0.10 | down |
| Malic acid | 1.09 | 0.02 | 0.06 | insig |
| D-(-)-ribofuranose | 1.08 | 0.05 | 0.10 | insig |
| Pentasiloxane | 1.08 | 0.03 | 0.08 | insig |
| Scyllo-inositol | 1.08 | 0.03 | 0.07 | insig |
| Lactic acid | 1.07 | 0.03 | 0.08 | insig |
| 9-octadecenoic acid | 1.06 | 0.07 | 0.13 | insig |
| N-acetyl-D-glucosamine | 1.06 | 0.07 | 0.13 | insig |
| Cholest-5-en-3-ol | 1.06 | 0.07 | 0.13 | insig |
| Butanedioic acid | 1.04 | 0.07 | 0.13 | insig |
| 4-aminobutanoic acid | 1.02 | 0.05 | 0.10 | down |
| Phthalic acid | 1.00 | 0.08 | 0.15 | insig |
