# Supplementary material for: Comparison of the gut microbiota and metabolism in different regions of Red Swamp Crayfish (Procambarus clarkii)
Source: Front Microbiol. 2023 Dec 22;14:1289634. doi: 10.3389/fmicb.2023.1289634 (PMC10770849; doi:10.3389/fmicb.2023.1289634)
Supplement: Supplementary file 7 [file Table_7.docx]

**Table S7 The difference in metabolites between the SD group and ZJ group.**

| Name | VIP | p_value | FDR | Type |
| --- | --- | --- | --- | --- |
| D-galactose | 1.33 | 0.00 | 0.01 | up |
| D-ribose | 1.32 | 0.00 | 0.12 | up |
| Tromethamine | 1.29 | 0.01 | 0.17 | up |
| Octanoic acid | 1.29 | 0.01 | 0.17 | up |
| Tetrasiloxane | 1.28 | 0.02 | 0.17 | up |
| 1,2,4-butanetriol | 1.28 | 0.01 | 0.17 | up |
| Methyl galactoside | 1.28 | 0.02 | 0.17 | down |
| Propanedioic acid | 1.27 | 0.00 | 0.12 | up |
| L-isoleucine | 1.26 | 0.03 | 0.17 | down |
| DL-phenylalanine | 1.26 | 0.03 | 0.17 | down |
| D-arabinose | 1.25 | 0.03 | 0.17 | down |
| Glyceryl-glycoside | 1.25 | 0.02 | 0.17 | down |
| 4-aminobutanoic acid | 1.25 | 0.03 | 0.17 | down |
| D-(+)-trehalose | 1.25 | 0.03 | 0.17 | down |
| L-serine | 1.25 | 0.03 | 0.17 | down |
| 9-octadecenoic acid | 1.25 | 0.03 | 0.17 | up |
| L-methionine | 1.24 | 0.03 | 0.17 | down |
| Decane | 1.24 | 0.01 | 0.17 | up |
| Mandelic acid | 1.23 | 0.03 | 0.17 | up |
| Pentasiloxane | 1.23 | 0.01 | 0.17 | up |
| Palmitic acid | 1.22 | 0.04 | 0.17 | down |
| L-aspartic acid | 1.22 | 0.05 | 0.17 | down |
| Tyrosine | 1.21 | 0.05 | 0.17 | down |
| Decanoic acid | 1.20 | 0.05 | 0.17 | insig |
| L-5-oxoproline | 1.20 | 0.05 | 0.17 | insig |
| Glycerol monostearate | 1.20 | 0.05 | 0.17 | down |
| Acetic acid | 1.20 | 0.05 | 0.17 | up |
| Putrescine | 1.20 | 0.04 | 0.17 | down |
| β-D-glucopyranose | 1.18 | 0.04 | 0.17 | down |
| 3-α-mannobiose | 1.18 | 0.07 | 0.19 | insig |
| Dodecanoic acid | 1.18 | 0.06 | 0.17 | insig |
| 5-dodecenoic acid | 1.16 | 0.06 | 0.18 | insig |
| Heptasiloxane | 1.15 | 0.07 | 0.19 | insig |
| Cholest-5-en-3-ol | 1.14 | 0.07 | 0.19 | insig |
| L-leucine | 1.14 | 0.03 | 0.17 | down |
| Pentanedioic acid | 1.13 | 0.08 | 0.21 | insig |
| Phthalic acid | 1.11 | 0.08 | 0.21 | insig |
| Glycine | 1.09 | 0.11 | 0.25 | insig |
| 9-decenoic acid | 1.09 | 0.10 | 0.24 | insig |
| Cholesterol | 1.07 | 0.06 | 0.17 | insig |
| Pyroglutamic acid | 1.07 | 0.12 | 0.27 | insig |
| Hexadecanoic acid | 1.06 | 0.11 | 0.25 | insig |
| Tridecanoic acid | 1.04 | 0.09 | 0.23 | insig |
